# Synthetic tetracycline-controllable shRNA targeting long non-coding RNA HOXD-AS1 inhibits the progression of bladder cancer

**DOI:** 10.1186/s13046-016-0372-5

**Published:** 2016-06-21

**Authors:** Jianfa Li, Chengle Zhuang, Yuchen Liu, Mingwei Chen, Yincong Chen, Zhicong Chen, Anbang He, Junhao Lin, Yonghao Zhan, Li Liu, Wen Xu, Guoping Zhao, Yinglu Guo, Hanwei Wu, Zhiming Cai, Weiren Huang

**Affiliations:** Key Laboratory of Medical Reprogramming Technology, Shenzhen Second People’s Hospital, Clinical Institute of Shantou University Medical College, First Affiliated Hospital of Shenzhen University, Shenzhen, 518039 Guangdong Province People’s Republic of China; Shantou University Medical College, Shantou, 515041 Guangdong Province People’s Republic of China; Guangdong and Shenzhen Key Laboratory of Male Reproductive Medicine and Genetics, Institute of Urology, Peking University Shenzhen Hospital, Shenzhen PKU-HKUST Medical Center, Shenzhen, 518036 People’s Republic of China; Anhui Medical University, Hefei, 230000 Anhui Province People’s Republic of China; Shanghai-MOST Key Laboratory of Health and Disease Genomics, Chinese National Human Genome Centerat Shanghai, Shanghai, 200000 People’s Republic of China; Department of Urology, Peking University First Hospital, Institute of Urology, Peking University, National Urological Cancer Center, Beijing, 100034 People’s Republic of China

**Keywords:** Bladder cancer, lncRNA, HOXD-AS1, Synthetic biology, Tetracycline-controllable shRNA

## Abstract

**Background:**

Long non-coding RNAs (lncRNAs) have been proved to act as key molecules in cancer development and progression. Dysregulation of lncRNAs is discovered in various tumor tissues and cancer cells where they can serve as oncogenes or tumor suppressors. Long non-coding RNA HOXD-AS (HOXD cluster antisense RNA 1) has recently been identified to be involved in the development of several cancers including neuroblastoma, adenocarcinomas and breast cancer. However, the role of HOXD-AS1 in bladder cancer remains unknown.

**Methods:**

The synthetic tetracycline-controllable shRNA was used to modulate the level of HOXD-AS1 by adding different concentrations of doxycycline (dox). RT-qPCR was used to detect the expression level of HOXD-AS1. Cell proliferation was determined by CCK-8 assay and EdU incorporation experiment when HOXD-AS1 was knocked down. We used wound-healing assay for detecting the effect of HOXD-AS1 on cell migration. Eventually, cell apoptosis was determined by caspase 3 ELISA assay and flow cytometry assay.

**Results:**

In this study, we found that the expression level of HOXD-AS1 was significantly increased in bladder cancer tissues and cells. Furthermore, high expression of HOXD-AS1 was significantly related to tumor size, histological grade and TNM stage. In vitro assays confirmed that knockdown of HOXD-AS1 suppressed cell proliferation/migration and increased the rate of apoptotic cell in bladder cancer cells. At last, we used the important element of synthetic biology, tetracycline(tet)-controllable switch, to construct tet-controllable shRNA vectors which can modulate the expression of HOXD-AS1 in a dosage-dependent manner.

**Conclusions:**

Our research suggested that high expression of HOXD-AS1 may be involved in the bladder cancer carcinogenesis through inhibiting the phenotypes and activating endogenous cancer-related molecular pathways. Therefore, HOXD-AS1 may act as an oncogene and provide a potential attractive therapeutic target for bladder cancer. In addition, the synthetic tetracycline-controllable shRNA may provide a novel method for cancer research in vitro assays.

## Background

Bladder cancer is a fatal disease with high morbidity and mortality. It is estimated that 683,310 cases have been diagnosed in the United States each year. It has become the fourth most common tumor among men with morbidity almost 4 times than that among women [1]. Approximately 70 % of patients present non-muscle invasive bladder cancer (NMIBC) and patients with muscle-invasive bladder cancer (MIBC) account for the rest [2, 3]. Transurethral resection of bladder tumor (TURBT) and intravesical chemotherapy usually have been chosen to treat NMIBC, while radical cystectomy is used for high-grade muscle-invasive bladder cancer (MIBC). However, the recurrence rate for NMIBC ranges from 50 to 70 % and 50 % of MIBC patients died within 5 years despite active treatment [4–7].

Long non-coding RNAs are widely defined as transcripts longer than 200 nucleotides without open reading frames [8, 9]. Although lncRNAs could not be translated into proteins, they function as oncogenes or tumor suppressors, and exert important roles in the progression of malignant tumors [10]. HOXD-AS1 contains eight exons and its transcript is a novel lncRNA. It is transcribed from the HOXD cluster on human chromosome 2q31.2 in an antisense manner. HOXD-AS1 is up-regulated in neuroblastoma, adenocarcinomas and breast cancer. Moreover, it is closely associated with the progression and prognosis of these cancers [11–14]. HOXD is the member of HOX clusters of genes which involves in regulating embryogenesis and organogenesis. Dysregulation of HOX genes have been found in various cancers. They act as vital regulators in the processes of methylation and histone modification [15, 16]. However, there is no report about the role of HOXD-AS1in bladder cancer.

Synthetic biology combines biological components and devices from different organisms to design and engineer artificial biological networks, pathways and systems that have novel functions and biomedical applications. Conveniently, these components can be created or obtained from different organisms [17–20]. Utilizing medical synthetic biology to fight against cancer has become a hot topic in recent years. Base on the principle and technology of synthetic biology, we used a standard method to synthesize tetracycline-controllable vectors which could modulate the expression of target gene in a dosage-dependent manner. This design provides a manageable research device to treat bladder cancer.

Here, we reveal the role of HOXD-AS1 in bladder cancer. Our data suggests that HOXD-AS1 may serve as an oncogene in the progression of bladder cancer, and a potential target for the bladder cancer treatments. Furthermore, the tetracycline-controllable shRNA can control the expression level of HOXD-AS1 in a dose-dependent manner and inhibit the development of bladder cancer cells, which may open up a novel way for treating bladder cancer.

## Methods

### Patients and tissue specimens

A total of 50 bladder cancer patients who underwent partial or radical cystectomy were included in this study. All specimens were stored in liquid nitrogen immediately after resection. The diagnosis was based on postoperative pathological analysis. The understanding of this study and written informed consent was received from each patient. The Institutional Review Board of Shenzhen Second People’s Hospital has approved this study.

### Cell culture

The human bladder cancer cells (5637 and T24) and the human normal bladder epithelial cells (SV-HUC-1) were obtained from the Institute of Cell Biology, Chinese Academy of Sciences (Shanghai, China). The 5637 cells were grown in RPMI-1640 Medium (Gibco), supplemented with 10 % fetal bovine serum (Gibco) at 37 °C in a 5 % CO_2_ humidified incubator. The T24 cells were cultured in Dulbecco’s modified Eagle’s medium (Gibco), supplemented with 10 % fetal bovine serum (Gibco) in a 5 % CO_2_ humidified incubator at 37 °C. SV-HUC-1 cells were cultured in F12K medium.

### Construction of synthetic tet-controllable shRNA targeting HOXD-AS1

HOXD-AS1 siRNA (si-HOXD-AS1) was synthesized by GenePharma, Suzhou, China according to a previous study. The sequence was GAAAGAAGGACCAAAGTAA [12]. Negative control siRNA (si-NC) was also provided from GenePharma. To construct tet-regulatable shRNAs, the company (FulenGen, Guangzhou, China) inserted the sequences of the associative tet-inducible shRNA targeting HOXD-AS1 and the negative control into tetracycline-inducible plasmid vectorspsi-LVRInU6TGP, respectively.

### Cell transfection

When the cells were grown to 40 % confluence in six-well plates, they were transfected with 200 pmol si-HOXD-AS1 or si-NC using Lipofectamine 2000 (Invitrogen, Carlsbad, CA) according to the instructions. However, the cells were transfected with 3 μg plasmid vectors (tet-controllable HOXD-AS1 shRNA or tet-controllable NC shRNA) when they got 70 % confluence.

### RNA extraction

Total RNA was obtained from the tissues or cells by using Trizol reagent (Invitrogen, Carlsbad, CA, USA) according to the instructions. cDNA was transcribed from 1 μg total RNA using Reverse Transcription Kit (Takara, Dalian, China). RT-qPCR was performed on the ABI7000 system (Applied Biosystems, Foster City, CA, USA). The reaction system contains 2ul of cDNA, 0.5 μl of forward primer, 0.5 ul of reverse primer, 5ul of qPCR Mix(TaKaRa) and 3ul of deionized water. The 2^–ΔΔCt^ method was chosen to calculate the relative expression levels. The expression of GAPDH was chosen as the endogenous control. The primer sequences were listed as follows: HOXD-AS1 primers forward 5’-GGCTCTTCCCTAATGTGTGG-3’, reverse: 5’-CAGGTCCAGCATGAAACAGA-3’; GAPDH primers forward 5’-CGCTCTCTGCTCCTCCTGTTC-3’, reverse: 5’- ATCCGTTGACTCCGACCTTCAC-3’.

### Cell proliferation assays

The CCK-8 assay (TransGen, Beijing, China) was used to examine cell proliferation. 5 × 10^3^ cells were seeded at 96-well plates at 37 °C for 24 h, and then transfected with either siRNA or plasmids. After 24, 48, 72 h, 10 μl CCK-8 was added to the plates and incubated at 37 °C for 1 h. The absorbance at 450 nm was detected by automatic microplate reader (Bio-Rad, Hercules, CA, USA). To intuitively observe the proliferative cells, EdU incorporation experiment was performed using an EdU assay kit (Ribobio, Guangzhou, China) according to the manufacturer’s instructions.

### Wound-healing assay

To explore the effect of HOXD-AS1 on migration in 5637 and T24 cells, we performed the wound-healing assay. At 48 h post-transfection, we used the yellow pipette tip to scratch a wound field. Then cells were grown in the medium without fetal bovine serum. The mobilized cells were observed by a digital camera system after 24 h and the migration distance was measured by the software program HMIAS-2000.

### Cell apoptosis assays

Cell apoptosis was determined by caspase 3 ELISA assay and flow cytometry assay. T24 and 5637 cells were transfected with siRNAs or plasmids. After incubation for 48 h, we used the the caspase 3 ELISA assay kit to detect the activity of caspase 3. A microplate reader was used to measure the OD values. In order to understand the accurate rate of apoptotic cellS, we used the flow cytometry assay. After transfection for 48 h, cells were collected and then washed by PBS. Besides, cells were mixed with Annexin V-FITC (AV) and Propidium Iodide (PI), and incubated in darkness for 15 min. The rate of apoptotic cells was measured by a flow cytometer (EPICS, XL-4, Beckman, CA, USA). Each experiment was repeated at least three times.

### Statistical analysis

Each experiment was performed in triplicate. All quantitative data were expressed as mean ± standard deviation (SD). Statistical analyses were performed by using SPSS 18.0 software (IBM). Statistical significance was tested by Student’s t-test, Chi square test or ANOVA. *P <* 0.05 was considered to be statistically significant.

## Results

### LncRNA HOXD-AS1 was up-regulated in bladder cancer tissues and cells, and positively correlated with tumor size, histological grade and TNM stage

**Fig. 1:**
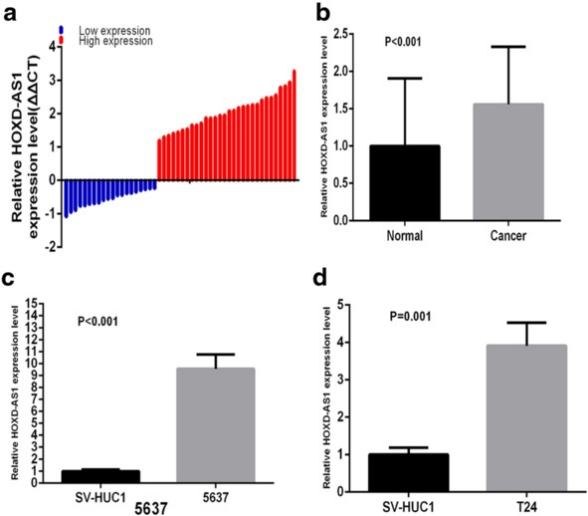
Relative HOXD-AS1 expression level in bladder cancer tissues and cells. **a** A total of 50 bladder cancer patients included in this study were divided into two groups: the relative low expression group (*n =* 20) and the relative high expression group (*n =* 30). **b** HOXD-AS1 was up-regulated in bladder cancer tissues compare to matched non tumor tissues (*P =* 0.002). **c**, **d** HOXD-AS1 expression level were higher in 5637 (*P <* 0.001) and T24 cells (*P =* 0.001) than normal human bladder epithelial cells (SV-HUC1)

The relative expression level of lncRNA HOXD-AS1 in 50 pairs of matched bladder cancer tissues was examined by performing real-time qPCR. HOXD-AS1 was significantly up-regulated in 60 % (30 of 50) of bladder tumor tissues (*P <* 0.001, Fig. 1a, b). Compared with the normal bladder epithelial cell line SV-HUC1,HOXD-AS1 was expressed at a higher level in bladder cell lines (*P <* 0.001 in 5637, *P =* 0.001 in T24, Fig. 1c, d). These results suggest that HOXD-AS1 may function as an oncogene in bladder cancer. We further analyzed the relationship between HOXD-AS1 and patients’ clinical pathologic factors. As shown in Table 1, high HOXD-AS1 expression was highly correlated with tumor size (*P =* 0.006), histological grade (*P =* 0.044), and TNM stage (*P =* 0.009). However, high HOXD-AS1 expression was irrelevant with the parameters including gender, age and lymphatic metastasis. Taken together, these data indicated that HOXD-AS1 may act as an oncogene in the progression of bladder cancer.

**Table 1:** Correlation between HXOD-AS1 expression and clinical characteristics of bladder cancer patients

Parameters	Group	Total	HOXD-AS1 expression		*P* value
			High(*n =* 30)	Low(*n =* 20)	
Age(years)	<60	31	17	14	0.768
	>60	19	12	7	
Gender	Male	36	19	17	0.537
	Female	14	9	5	
Tumor size (cm)	<3 cm	18	6	12	0.006
	≥3 cm	32	24	8	
Histological grade	Low	22	9	13	0.044
	Hight	28	20	8	
TNM stage	I	29	9	20	0.009
	II/III/IV	21	15	6	
Lymphatic metastasis	N0	44	29	15	0.650
	N1 or above	6	5	1	

*P <* 0.05 was considered significant. (Chi-square test was performed)

### Knockdown of HOXD-AS1 inhibited bladder cancer cell proliferation

**Fig. 2:**
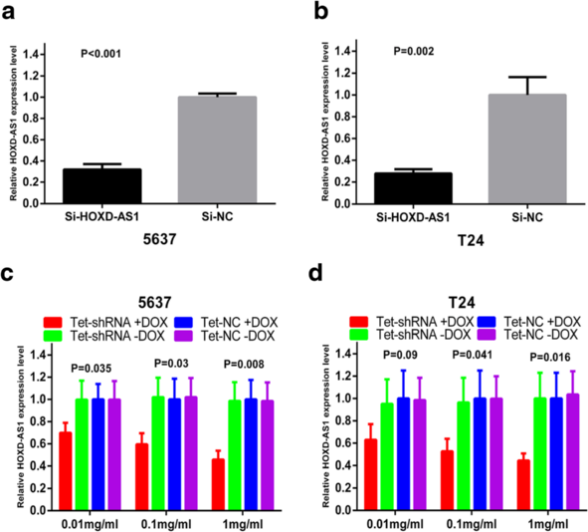
Expression changes of HOXD-AS1 after transfection of siRNA or tet-shRNA. **a**, **b** The si-HOXD-AS1 significantly reduced the expression level of HOXD-AS1 in 5637 (*P <* 0.001) and T24 (*P =* 0.002). **c**, **d** The tet-shRNA HOXD-AS1 induced by dox regulated the expression of HOXD-AS1 in a dosage-dependent manner in 5637 (*P =* 0.008) and T24 cells (*P =* 0.016)

**Fig. 3:**
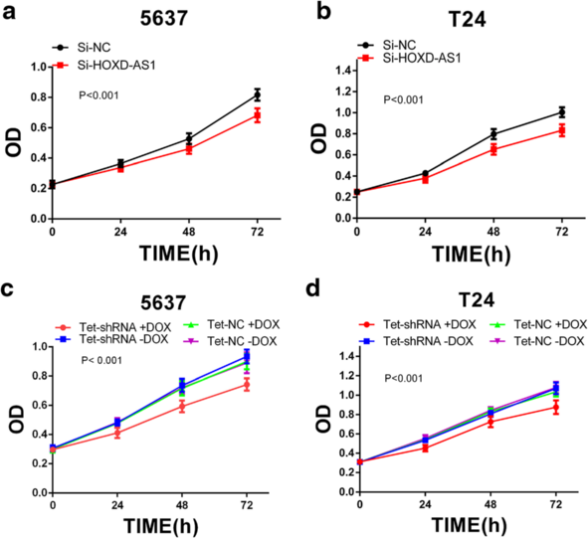
Knockdown of HOXD-AS1 by siRNA or tet-shRNA inhibits cell proliferation. CCK-8 assay was performed to investigate the cell growth curve. **a**, **b** Cell proliferation inhibition was detected in 5637 (*P <* 0.001) and T24 cells (*P <* 0.001) after transfection with si-HOXD-AS1 (**c**, **d**) The tet–shRNA induced by 1ug/ml dox significantly inhibits cell proliferation in 5637 (*P <* 0.001) and T24 cells (*P <* 0.001). Data were expressed as means ± SD from three independent experiments

**Fig. 4:**
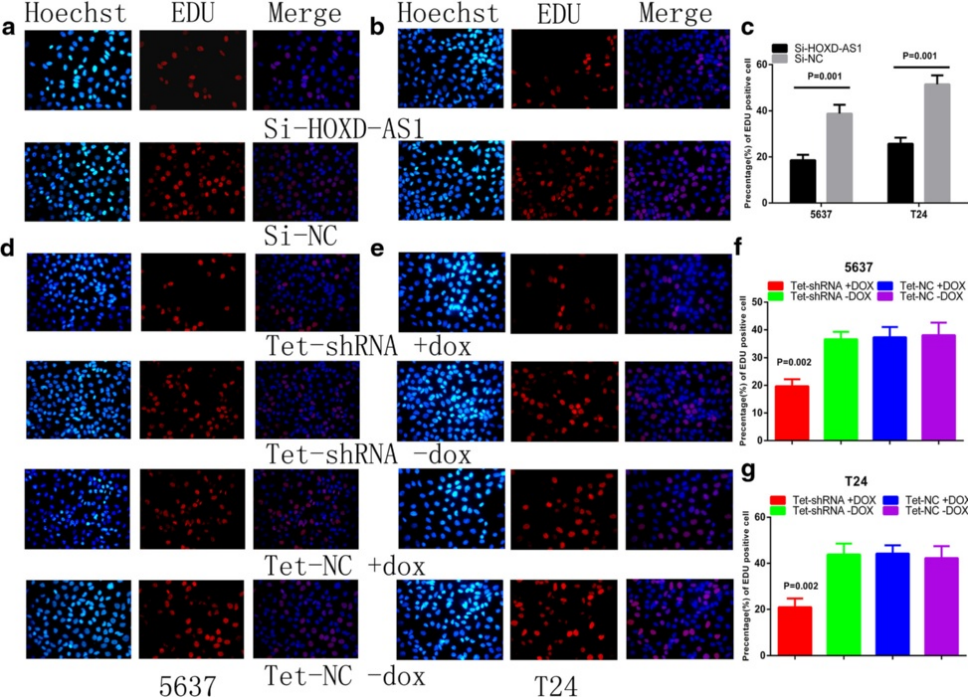
The multiplication capacity of 5637 and T24 cells was declined after transfection with si-HOXD-AS1 or tet-shRNA. EdU assay was performed. **a**, **b** The number of EdU positive cells was decreased significantly after transfected with si-HOXD-AS1 in 5637 and T24 cells. **c** The percentage of EDU positive cells in 5637 (*P =* 0.001) and T24 cells (*P =* 0.001) can be seen. **d**, **e** The number of EdU positive cells was decreased significantly after transfection with tet-shRNA which was induced by 1ug/ml dox. **f**, **g** The percentage of EdU positive cells in 5637 (*P =* 0.002) and T24 cells (*P =* 0.002) can be seen. Data were expressed as means ± SD from three independent experiments

5637 and T24 cells were transfected with si-HOXD-AS1 or si-NC to investigate whether HOXD-AS1 promotes proliferation of bladder cancer cells. The relative expression level of HOXD-AS1 was detected by RT-qPCR at 48 h after transfection. As shown in Fig. 2a, b, the relative level of HOXD-AS1 in bladder cancer cells was significantly decreased by si-HOXD-AS1, indicating that si-HOXD-AS1 transfection was successful (*P <* 0.001 in 5637, *P =* 0.002 in T24). The relative expression level of HOXD-AS1 was reduced by 68.05 % in 5637 cells and 72.08 % in T24 cells. Next, we performed the CCK-8 assay to explore the role of si-HOXD-AS1 in cell proliferation. As shown in Fig. 3a, b, the decreased ability of proliferation was obviously seen in 5637 and T24 cells. Furthermore, the percentage of EdU positive cells was decreased significantly after HOXD-AS1 knockdown (Fig. 4a, b). The percentage of EdU positive cells in si-HOXD-AS1 group was decreased by 20.29 % in 5637 (*P =* 0.001) and 25.76 % in T24 (*P =* 0.001) (Fig. 4c). All these data suggest that high expression of HOXD-AS1 promoted the proliferation of bladder cancer cells.

### Effects of HOXD-AS1 on cell migration in bladder cancer cells

**Fig. 5:**
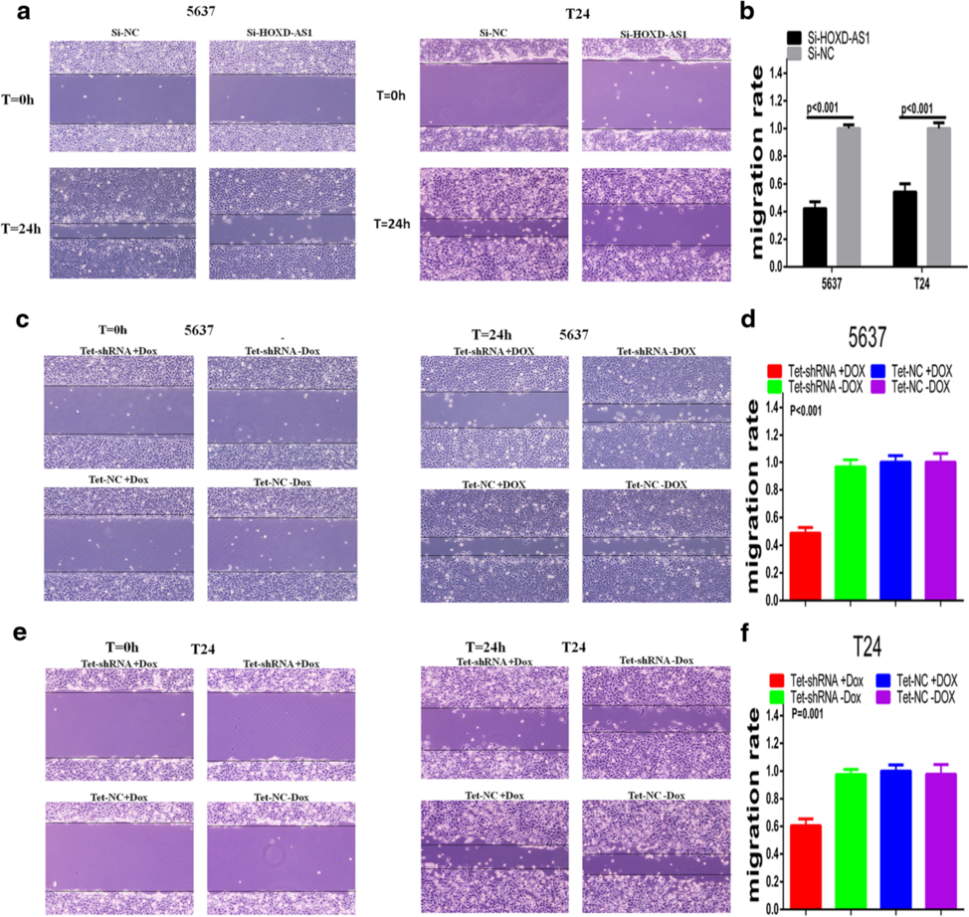
Silencing HOXD-AS1 inhibited cell migration. **a** 5637 and T24 cells transfected with si-HOXD-AS1 showed decreased migration capacity compare with si-NC transfected cells. **b** The migration rate of 5637 (*P <* 0.001) and T24 cells (*P <* 0.001) is shown in the bar chart. **c**, **e**. The tet-shRNA induced by 1ug/ml dox significantly inhibited the migration ability of 5637 and T24. **d**, **f** The migration rate of 5637 (*P <* 0.001) and T24 cells (*P =* 0.001) transfected with tet-shRNA were observed. Data were expressed as means ± SD from three independent experiments

As shown in Fig. 5a, compared with si-NC group, the migration capacity was significantly decreased in si-HOXD-AS1 group. As shown in Fig. 5b, the relative migration rate in the si-HOXD-AS1 group was declined by 57.83 % in 5637 cells (*P <* 0.001) and 45.79 % in T24 cells (*P <* 0.001). These suggest that silencing HOXD-AS1 weakened the capacity of migration in bladder cancer cells.

### Knockdown of HOXD-AS1 induced apoptosis

**Fig. 6:**
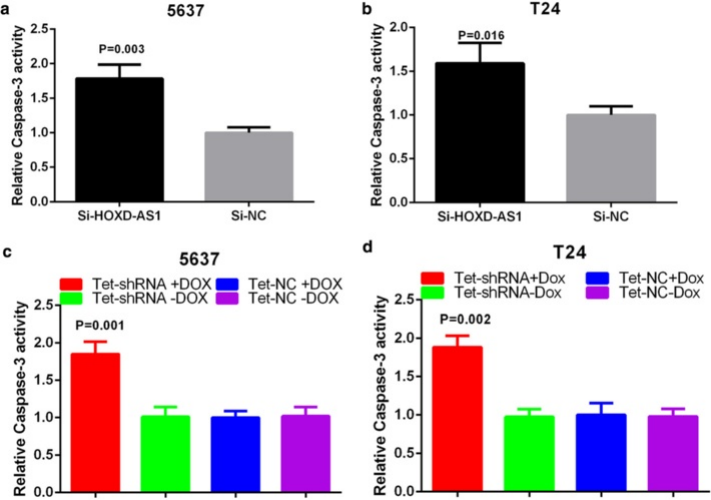
Knockdown of HOXD-AS1 promotes apoptosis using ELISA assay. **a**, **b** Relative activities of caspase-3 in 5637 (*P =* 0.003) and T24 (*P =* 0.016) were shown after transfection with si-HOXD-AS1. **c**, **d** The activity of caspase-3 was increased significantly after transfection with tet-shRNA in 5637 (*P =* 0.001) and T24 cells (*P =* 0.002). Results represent the mean ± SD from three independent experiments

**Fig. 7:**
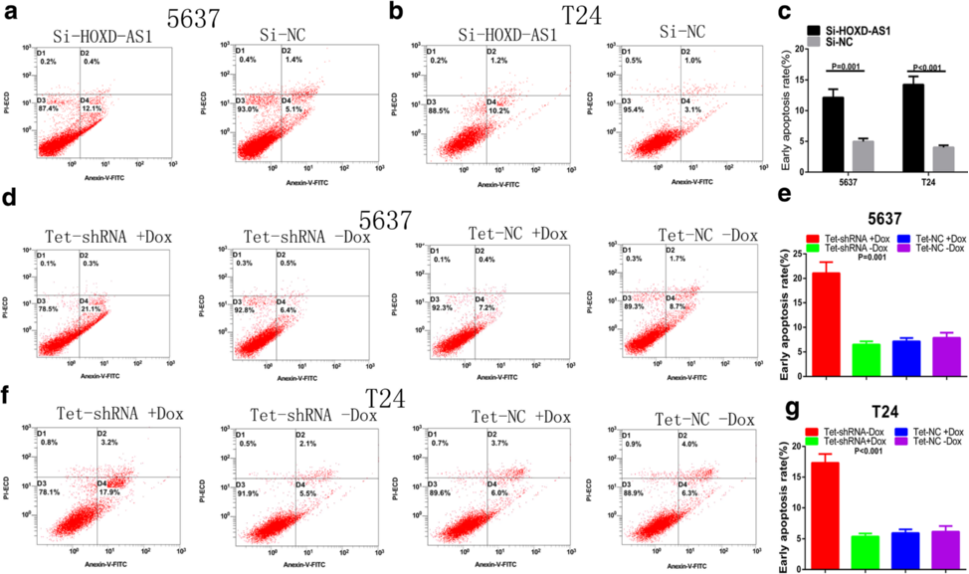
Transfection with si-HOXD-AS1 or tet shRNA induced apoptosis in 5637 and T24. **a**-**c** The rate of early apoptotic 5637 (*P =* 0.001) and T24 cells (*P <* 0.001) were increased significantly after transfection with si-HOXD-AS1. **d**-**g** Increased apoptotic cells were observed in tet-shRNA-transfected 5637 (*P =* 0.001) and T24 cells (*P <* 0.001). Results represent the mean ± SD from three independent experiments

Besides, we assume that knockdown of HOXD-AS1 may induce apoptosis. The detection of apoptosis was performed by caspase 3 ELISA assay and flow cytometry assay. As shown in Fig. 6a, b, the activities of caspase 3 in bladder cancer cells were significantly increased after transfected with si–HOXD-AS1 (*P =* 0.003 in 5637 and *P =* 0.016 in T24). What’s more, the ratio of apoptosis and early apoptosis fraction in si-HOXD-AS1 transfected cells were greater than the si-NC transfected cells (Fig. 7a-c, *P =* 0.001 in 5637 and *P <* 0.001 in T24). All these data indicate that high expression of HOXD-AS1 inhibited cell apoptosis in bladder cancer cells.

### Tet-controllable HOXD-AS1 shRNA induced by dox surpressed the expression of HOXD-AS1

Cells were transiently transfected with the plasmids (3 ug) which expressed the tet-controllable HOXD-AS1 shRNA or the negative control, respectively. After transfection, different concentrations of dox (0.01 mg/ml, 0.1 mg/ml and 1 mg/ml) were added to the cells. As shown in Fig. 2c, d, the tet-controllable HOXD-AS1 shRNA inhibit the relative expression level of HOXD-AS1 in a dose-dependent manner. When 1 mg/ml dox was added into vectors transfected 5637 and T24 cells, the expression level of HOXD-AS1 was decreased by 54.15 % in 5637 cells (*P =* 0.008) and 55.54 % in T24 cells (*P =* 0.016). Therefore, we chose 1 mg/ml as the best concentration for further study. These results confirmed that the tet-controllable HOXD-AS1 shRNA expressed in bladder cancer cells and inhibited the expression of HOXD-AS1.

### Tet-controllable HOXD-AS1 shRNA inhibited cell proliferation

To investigate the role of tet-controllable HOXD-AS1 shRNA on cell proliferation in bladder cancer cells, we performed the CCK-8 and EdU assays. CCK-8 assay showed that tet-controllable HOXD-AS1 shRNA induced by dox (1 mg/ml) inhibited cell proliferation significantly compared with the negative control (Fig. 3c, d). EdU assay came up with the same results as excepted (Fig. 4d, e). The percentage of EdU positive cells was reduced by 17.62 % in 5637 cells (*P =* 0.002) and 23.26 % in T24 cells (*P =* 0.002) (Fig. 4f, g). These results confirmed that tet-controllable HOXD-AS1 shRNA induced by dox could significantly inhibit cell proliferation.

### Tet-controllable HOXD-AS1 shRNA inhibited cell migration

Wound-healing assay was performed to confirm the effect of tet-controllable HOXD-AS1 shRNA on the migration ability in bladder cancer cells. As shown in Fig. 5c-f, the cells in tet-controllable HOXD-AS1 shRNA group displayed less migration ability. Compared with the negative control group, the migration rate of the tet-controllable HOXD-AS1 shRNA group was decreased by 51.2 % (*P <* 0.001)in 5637 cells and 39.41 % (*P =* 0.001) in T24 cells. These results indicated that tet- controllable HOXD-AS1 shRNA could strongly inhibit the migration ability of bladder cancer cells.

### Tet-controllable HOXD-AS1 shRNA induced cell apoptosis

Finally, we investigated the possible effect of tet-controllable HOXD-AS1 shRNA on the apoptosis of bladder cancer cells. Elisa assay was performed to determine the relative activity of caspase-3. As shown in Fig. 6c, d, the relative activity of caspase-3 was increased obviously in the tet-controllable HOXD-AS1 shRNA group (*P =* 0.001 in 5637 and *P =* 0.002 in T24). Furthermore, we performed the flow cytometry assay to explore the apoptosis ratio. As shown in Fig. 7d-g, the ratio of apoptosis and early apoptosis fraction in the tet-controllable HOXD-AS1 shRNA group were increased significantly. Results suggest that the tet-controllable HOXD-AS1 shRNA could induce apoptosis in bladder cancer cells (*P =* 0.001 in 5637 and *P <* 0.001 in T24).

## Discussion

Bladder cancer is a common malignancy, which has brought great economic burden to the world. The conventional therapy, such as surgery, radiotherapy and chemical therapy can not improve 5 year survival rate obviously [21]. Lots of literatures show that numerous lncRNAs involve in the process of tumorigenesis and tumor progression [22–24]. What’s more, lncRNAs may serve as tumor markers and therapeutic targets, such as PVT1, Xist, ATB and MALAT1 [25–28]. HOXD-AS1 is transcribed from the HOXD gene cluster between HOXD1 and HOXD3. Besides, HOXD-AS1 is closely related to HOXD3 expression [11]. High HOXD-AS1 levels are associated with neuroblastoma progression and regulated by the PI3K/Akt pathway which has been proved to promote oncogenesis via inhibiting cell apoptosis [29]. Furthermore, a series of genes are controlled by HOXD-AS1 and these genes may serve as biomarkers of neuroblastoma recurrence and affect the patients’ survival [12]. HOXD-AS1 was also found to be overexpressed in adenocarcinomas and breast cancer, and is characterized as the indicator of poor prognosis [13, 14]. According to our knowledge, the role of HOXD-AS1 in bladder cancer is still unclear. So, it is necessary to detect the biological function of HOXD-AS1 in bladder cancer cells.

This is the first study to investigate the function of HOXD-AS1 in bladder cancer. In this research, we found that HOXD-AS1 was up-regulated in bladder cancer tissues and cells, indicating that HOXD-AS1 may act as an oncogene. Further experiments demonstrate that HOXD-AS1 may be viewed as a promising tumor maker. High expression of HOXD-AS1 was positively associated with clinicopathologic features, including tumor size, histological grade and TMN stage. To get a better insight to the function of HOXD-AS1, we silenced HOXD-AS1 with specific siRNA and found that inhibition of HOXD-AS1 could inhibit cell proliferation, suppress cell migration and increase apoptosis in bladder cancer cells. Significantly, it is the first research to verify the oncogenic role of HOXD-AS1 in bladder cancer. Further studies on the modulation of HOXD-AS1 expression and more thorough molecular mechanisms are needed.

With the rapid development of synthetic biology, more synthetic gene networks are applied in medical research, including immuno-regulatory networks and cancer therapy gene circuits [30–32]. Synthetic biological gene switches including ABA transcriptional switches, tet-on post-transcriptional switches and hoechst aptamer translational switches form the basis to engineer novel and complex gene networks [33–35]. Synthetic tet-on switch is commonly accepted and is employed to control genes expression [36, 37]. Inspired by these previous works, we constructed the synthetic tetracycline-controllable shRNA targeting HOXD-AS1 and detected its anti-cancer effects in bladder cancer cells. Our data demonstrated that the synthetic tetracycline-controllable shRNA targeting HOXD-AS1 effectively suppressed the expression level of HOXD-AS1 in a dose-dependent manner, which subsequently inhibited the ability of proliferation/migration and induced apoptosis in bladder cancer cells. We suppose that the synthetic tetracycline-controllable shRNA could control multiple genes expression at the same time and increasing the transfection efficiency could improve the efficiency of this device.

It should be noted that there seems to be no significant decrease in proliferation between 48 and 72 h in both T24 and 5637 cells. But the apoptosis assay shows that there is a significant increase at 48 h. One possible explanation for these contradictory results is that the change in proliferation rate may not be readily reflected by the rate of increase of OD value when the cell number was continuously increased.

## Conclusions

In conclusion, our research suggested that HOXD-AS1 may get involved in the bladder cancer carcinogenesis by activating endogenous cancer-related molecular pathways. Therefore, HOXD-AS1 may become a potential and promising therapeutic target. Furthermore, we use the synthetic biology component to construct the synthetic tetracycline-controllable shRNA for controlling the expression of HOXD-AS1 artificially and inhibiting the progression of bladder cancer cells. Maybe, it can provide a novel approach for effectively suppressing specific oncogenes and promote the development of new anti-cancer method in the area of medical synthetic biology. This work may bring a promising future to fight against bladder cancer.
