# Correction: Synthetic tetracycline-controllable shRNA targeting long non-coding RNA HOXD-AS1 inhibits the progression of bladder cancer

**DOI:** 10.1186/s13046-023-02908-6

**Published:** 2023-11-28

**Authors:** Jianfa Li, Chengle Zhuang, Yuchen Liu, Mingwei Chen, Yincong Chen, Zhicong Chen, Anbang He, Junhao Lin, Yonghao Zhan, Li Liu, Wen Xu, Guoping Zhao, Yinglu Guo, Hanwei Wu, Zhiming Cai, Weiren Huang

**Affiliations:** 1 Guangdong Province, Key Laboratory of Medical Reprogramming Technology, Shenzhen Second People’s Hospital, Clinical Institute of Shantou University Medical College, First Affiliated Hospital of Shenzhen University, Shenzhen, 518039 People’s Republic of China; 2 Guangdong Province, Shantou University Medical College, Shantou, 515041 People’s Republic of China; 3 Guangdong and Shenzhen Key Laboratory of Male Reproductive Medicine and Genetics, Institute of Urology, Peking University Shenzhen, Hospital, Shenzhen PKU-HKUST Medical Center, Shenzhen, 518036 People’s Republic of China; 4 Anhui Province People’s, Anhui Medical University, Hefei, 230000 Republic of China; 5 Shanghai-MOST Key Laboratory of Health and Disease Genomics, Chinese National Human Genome Center at Shanghai, Shanghai, 200000 People’s Republic of China; 6 Department of Urology, Peking University First Hospital, Institute of Urology, Peking University, National Urological Cancer Center, Beijing, 100034 People’s Republic of China

**Correction:** ***J Exp Clin Cancer Res*** **35, 99 (2016)**

**https://doi.org/10.1186/s13046-016-0372-5**

Following publication of the original article [1], author found errors in Fig. 7, specifically:

- Fig. 7d – the result of flow cytometry assay in Tet-shRNA + Dox group was misplaced
- Fig. 7f – the results of flow cytometry assay in Tet-shRNA –Dox group and Tet-NC–Dox group were misplaced

**Fig. 7:**
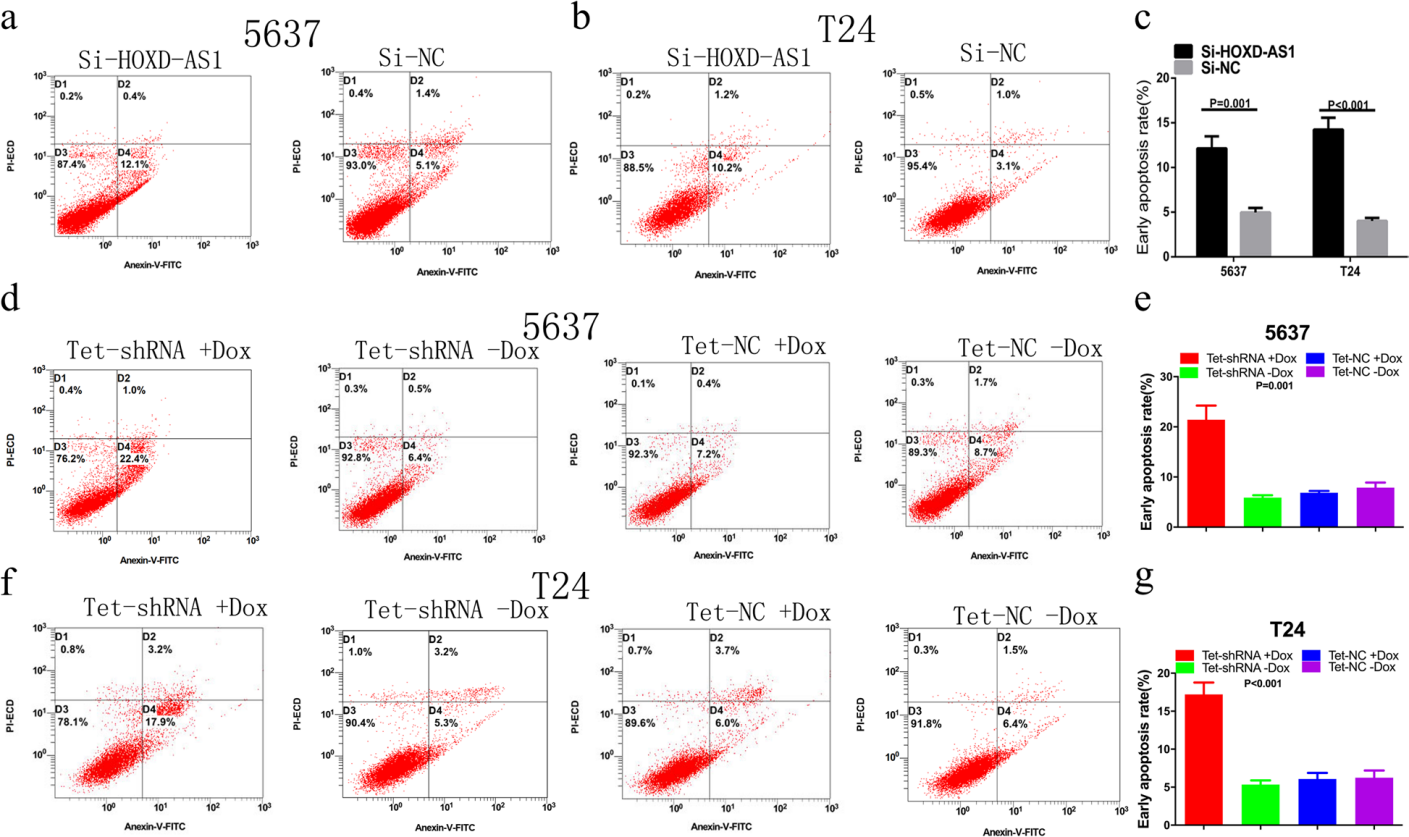
Transfection with si-HOXD-AS1 or tet shRNA induced apoptosis in 5637 and T24. **a**-**c** The rate of early apoptotic 5637 (*P* = 0.001) and T24 cells (*P* < 0.001) were increased significantly after transfection with si-HOXD-AS1. **d**-**g** Increased apoptotic cells were observed in tet-shRNA-transfected 5637 (*P* = 0.001) and T24 cells (*P* < 0.001). Results represent the mean ± SD from three independent experiments

The corrected Fig. 7 is given here. The correction does not affect the conclusions of the article.
